# Features and functionality of the Iterapi platform for internet-based psychological treatments: An update

**DOI:** 10.1016/j.invent.2026.100955

**Published:** 2026-05-23

**Authors:** George Vlaescu, Per Carlbring, Gerhard Andersson

**Affiliations:** a Department of Behavioural Sciences and Learning, Linköping University, Linköping, Sweden; b Department of Psychology, Stockholm University, Stockholm, Sweden; c School of Psychology, Korea University, Seoul, South Korea; d Department of Health, Education and Technology, Luleå University of Technology, Sweden; e Lusófona University, HEI-Lab: Digital Human-Environment Interaction Labs, Lisboa, Portugal

**Keywords:** Internet-based cognitive behaviour therapy, ICBT, Digital mental health, Treatment platform, Artificial intelligence, Iterapi

## Abstract

Internet-based cognitive behaviour therapy (ICBT) is now a well-established treatment modality supported by numerous controlled trials. Platforms for delivering these interventions have become critical infrastructure for both research and clinical practice, yet platform descriptions remain rare in the literature. In this article, we provide an updated description of the Iterapi platform, originally reported by Vlaescu et al., 2016. Over the past decade, Iterapi has been used in more than 100 published research studies, spanning over 35 clinical conditions (including depression, anxiety disorders, insomnia, tinnitus, loneliness, and climate anxiety) across at least 20 countries on five continents. We describe the platform's core architecture and functionality, including treatment modules, questionnaire systems, communication tools, and automation features. We also report on significant developments since 2016, including enhanced security and regulatory compliance (GDPR, data protection impact assessments), new features for ecological momentary assessment, electronic identification and incorporation of artificial intelligence. Finally, we outline future directions including mobile application development, digital phenotyping, just-in-time adaptive interventions, and the role of emerging regulatory frameworks. This update is intended as a practical resource for researchers and clinicians considering platform-based delivery of internet interventions.

## Highlights

• Iterapi has supported 100+ studies across 35+ conditions in 20+ countries.
• Cultural adaptation studies demonstrate reach to immigrant populations.
• Legal requirements such as General Data Protection Regulation (GDPR) are handled by the system.
• Artificial intelligence solutions are in progress.

## 1 Introduction

The field of internet-based psychological treatment has changed considerably since the earliest trials in the late 1990s (Andersson, 2025). What began as bibliotherapy delivered via static web pages has developed into interactive platforms supporting multimedia content, real-time communication, automated assessment, and, increasingly, artificial intelligence (AI) components (Andersson, 2025). The evidence base has grown (Käll et al., 2024). Systematic reviews and meta-analyses now consistently demonstrate that therapist-guided ICBT produces outcomes equivalent to face-to-face cognitive behaviour therapy (CBT) for a range of psychiatric and somatic conditions (Hedman-Lagerlöf et al., 2023), with additional advantages in cost-effectiveness (Kählke et al., 2022), accessibility, and scalability (Vernmark et al., 2024).

Currently the broader digital mental health field has expanded rapidly, with smartphone apps, generative AI, and virtual reality emerging as complementary modalities (Löchner et al., 2025). This growth has been accompanied by a proliferation of platforms for delivering internet-based interventions. Some are commercially developed products designed for healthcare systems, such as SilverCloud (now part of Amwell), Minddistrict, and Deprexis. Others have emerged from academic research groups, such as THIS WAY UP and MindSpot in Australia, and Iterapi in Sweden. The platforms differ substantially in their design philosophy, degree of researcher control, data handling practices, and openness, differences affecting both clinical delivery and the methodological rigour and reproducibility of the research conducted on them (Vlaescu et al., 2016). Yet despite the central role that platforms play in internet intervention research, detailed descriptions of their features, architecture, and design rationale remain scarce in the literature.

The Iterapi platform was developed at Linköping University, Sweden, by the Internet, Health and Clinical Psychology Research Group in collaboration with the university's IT department. Since its original description (Vlaescu et al., 2016), the platform has been used extensively. A Scopus search conducted in March 2026 identified 167 publications mentioning Iterapi, of which at least 112 report results from studies that used the platform for treatment delivery or data collection. This figure is likely conservative, as it excludes book chapters, theses, and outlets not indexed in Scopus, and does not capture studies that describe the platform without specifically naming it. These studies span more than 35 clinical conditions, including depression, social anxiety disorder, generalised anxiety disorder, panic disorder, insomnia, chronic pain, tinnitus, perfectionism, procrastination, loneliness, climate change distress, post-traumatic stress, eating disorder prevention, caregiver burden, cardiovascular disease comorbidity, problem gambling, assertiveness, and sport psychology, and have been conducted in at least 20 countries across five continents. Peak publication output occurred in 2021, with 20 papers, likely due to both accumulated momentum from ongoing projects and the demand for digital mental health services during the COVID-19 pandemic (De Witte et al., 2021).

The aim of this paper is to provide an updated description of the Iterapi platform, reporting on new features and functionality added since 2016, positioning the platform among current digital mental health tools, and describing recent work on AI integration. We also address security and regulatory compliance developments, particularly in light of the European Union's General Data Protection Regulation (GDPR) and the emerging AI Act. Our aim is to provide a practical resource for researchers and clinicians evaluating platforms for internet-based interventions.

## 2 About the Iterapi platform

Iterapi is a web-based platform designed primarily to deliver guided and unguided psychological interventions in a research context. The platform integrates content management (treatment materials, public information pages), user management (administrators, therapists, participants, and user grouping), assessment tools (questionnaires with automatic scoring and scheduling), communication systems (asynchronous messaging, live chat, SMS), and data export facilities into a unified interface. While its primary use has been for internet-based CBT, Iterapi has also supported psychodynamic therapy (Mechler et al., 2024), acceptance and commitment therapy (Ivanova et al., 2016), mindfulness-based interventions (Dumarkaite et al., 2021) and virtual reality exposure studies (Lindner et al., 2020).

A distinctive feature of Iterapi is that is hosted in a university setting. The platform is developed and maintained within the university infrastructure, with servers physically located at Linköping University (Sweden). This means that the research group retains full control over the platform's functionality, data handling, and development priorities, a level of transparency that distinguishes Iterapi from commercial platforms where the underlying code and data processing pipelines may be proprietary.

**Fig. 1:**
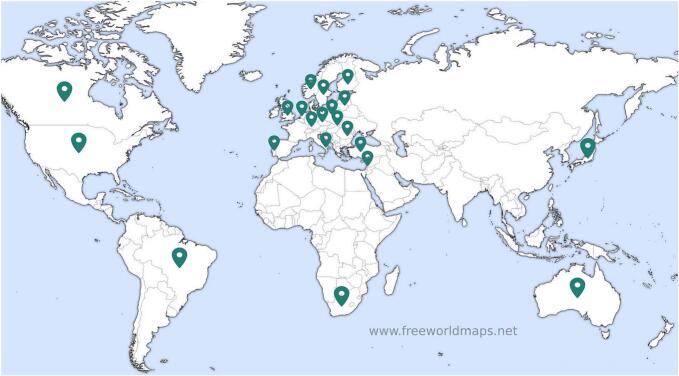
The countries in which Iterapi has been used. (Original world map image downloaded from www.freeworldmaps.net).

Each study on the platform operates as an independent instance with its own homepage, layout, database, and URL. This architectural choice ensures clear separation of data between studies and allows researchers to customise the platform's appearance and configuration for each trial. The platform interface has been translated into more than 20 languages, including Arabic, Czech, Dutch, Finnish, German, Hebrew, Italian, Japanese, Kurdish, Lithuanian, English, Persian, Polish, Portuguese, Romanian, Russian, Slovak, Spanish, Swedish, and Turkish, enabling collaborations with research groups in Brazil, Canada, Germany, Israel, Japan, Lithuania, Poland, Portugal, Romania, Turkey, the United Kingdom, the United States, Australia, Italy, Luxembourg, and many more. The countries in which Iterapi has been used is displayed in Fig. 1.

## 3 Installation, security, and compliance

### 3.1 Hosting and infrastructure

The platform is hosted on servers located at Linköping University, where hardware, operating system, and network infrastructure are provided by the university's IT department. The server environment is based on Linux, installed in a redundant physical configuration. The operating system and software packages are automatically updated and monitored by system administrators. Daily backups are performed and stored on servers located in a separate building from the production environment, ensuring data recovery in the event of hardware failure or other incidents.

### 3.2 Encryption and data protection

Given that most participants present with psychological problems, strict security practices are essential. All sensitive user information is stored encrypted in the databases using AES-256 with secret keys. It is not possible to establish a link between the stored encrypted data and identifiable individuals through database access alone. Data communication between the platform and users is encrypted using HTTPS/TLS. A professional security team within the university's IT department ensures that both the servers and the surrounding infrastructure meet government-level quality assurance requirements.

### 3.3 GDPR compliance

The platform's operations fall under the European Union's General Data Protection Regulation (GDPR; Regulation 2016/679), which has been in effect since May 2018. Linköping University, as the data controller for studies conducted at the institution, has implemented comprehensive GDPR compliance measures for the Iterapi platform. These include data protection impact assessments (DPIAs) conducted prior to new studies, clear legal bases for data processing (typically informed consent for research, in accordance with Article 6(1)(a) and Article 9(2)(a) of the GDPR), data minimisation principles, defined data retention periods, and procedures for handling data subject requests (access, rectification, erasure). For international collaborations, data processing agreements specify responsibilities and safeguards, particularly when studies involve participants or researchers outside the European Economic Area.

### 3.4 The European Health Data Space

The European Health Data Space Regulation (EHDS; Regulation 2025/327), adopted in early 2025, will reshape how health data, including data from digital interventions, can be used across the EU. The EHDS establishes rules for both primary use (facilitating continuity of care across borders) and secondary use (enabling research, innovation, and policy-making using health data). For a platform like Iterapi that handles health data across European borders in collaborative research projects, the EHDS will introduce new requirements and opportunities: harmonised data access procedures could simplify cross-national research collaborations, while secondary use provisions could enable more systematic use of aggregated treatment data for improving intervention design. The full implementation timeline extends through 2031, and the interaction between EHDS, GDPR, and the AI Act will need to be worked out in practice.

### 3.5 Implications of the EU AI Act

Regulatory developments can also have unexpected consequences for digital mental health research. A recent Iterapi-based trial of internet-delivered psychodynamic therapy for adolescent depression had to be discontinued after recruiting only 35 of a planned 240 participants, primarily because EU regulations prohibiting profiling-based online advertising for minors eliminated access to previously effective social media recruitment channels (Philips et al., 2026). This experience points to the need for new recruitment infrastructures that comply with evolving data protection laws while maintaining feasibility.

The European Union's Artificial Intelligence Act (Regulation 2024/1689), which entered into force in August 2024 with provisions being phased in through 2027, introduces a risk-based regulatory framework for AI systems. For platforms like Iterapi that are beginning to integrate AI components into psychological treatment delivery, the classification of such systems (likely as high-risk given their use in healthcare) will carry obligations regarding transparency, data governance, human oversight, and documentation.

### 3.6 Anonymity and the dark web

Some internet interventions require a high degree of anonymity for participants, dictated by the nature of the intervention or participant preferences. This means that user-identifiable information (including personal data, contact information, and internet location data such as IP addresses) must not be transmitted to or stored on the server.

Iterapi has made this possible by supporting deployment as a hidden service on the Tor network, where through specific server configurations, users' internet locations are not exposed to the servers. This capability has been successfully used in three studies, most notably the Prevent It trial, a randomised placebo-controlled study of internet-delivered CBT for individuals who use child sexual abuse material, which recruited participants globally from Darknet forums (Lätth et al., 2022). The Prevent It study represents, to our knowledge, the first RCT ever conducted entirely on the Darknet, demonstrating both the technical feasibility and the clinical potential of providing evidence-based interventions to populations that cannot be reached through conventional channels. Extensive development was required to ensure that the platform functions fully without JavaScript, which anonymous users typically disable for security reasons. This includes all interactive features such as chat, worksheets, and conditional content display.

## 4 The process flow of a study

Based on extensive experience running treatment studies over the internet (Andersson, 2025), we have developed a standard process flow that can be adapted to the needs of each study. This flow provides a useful overview for researchers and therapists new to internet-based interventions. The typical stages are presented in Table 1.

**Table 1:** Stages in a typical ICBT study.

Stage	Description
1. Initial setup	Choose a study name, layout, and logo. Configure the platform instance.
2. Staff accounts	Add administrator and therapist accounts. Staff can then begin working with the content.
3. Public homepage	Create and publish the study homepage used to advertise the study and inform potential participants.
4. Measures	Add questionnaires for screening, outcome assessment, and process measures. Many validated instruments are already available on the platform and can be exported to new studies.
5. Treatment content	Create the intervention programme with structured modules. Researchers typically do this themselves, with technical support for custom interactive exercises. It is common to adapt existing treatment materials (e.g., creating an adolescent version from an adult programme).
6. Recruitment	Advertise the study via social media, search engine advertisements, television, radio, newspapers, or bulletin boards, linking to the study homepage. During this stage, visitors register their interest by providing informed consent and completing screening questionnaires.
7. Evaluation and inclusion	Researchers analyse incoming registrations, typically conduct telephone interviews for diagnostic assessment and engagement, and hold inclusion meetings. For randomised controlled trials, participants are randomised to predefined study arms. This phase can also be performed automatically by the platform.
8. Treatment delivery	Included participants access treatment modules, typically one per week. Participants read materials and complete exercises; therapists review submissions and provide feedback.
9. Therapist communication	Throughout the treatment period, participants communicate with assigned therapists via asynchronous messaging, scheduled chat sessions, or other available channels.
10. Progress monitoring	In many studies, participants complete weekly outcome measures, allowing therapists to monitor progress and detect possible deterioration.
11. Post-treatment and follow-up	Participants complete post-treatment questionnaires. Follow-up assessments (e.g., at 6, 12, or 36 months) can be scheduled automatically by the platform.

While this represents the standard flow, many projects define their own procedures with additional steps, such as automatic booking of interview times, automatic exclusion based on screening results, and fully automated randomisation. Notably, at least two unpublished studies, one with over 2400 participants, have run entirely automatically, from registration through screening, randomisation, treatment delivery, and follow-up, with therapists intervening only when participants had questions or to moderate discussion forums.

## 5 Main functions and sections

### 5.1 Responsive design

The platform layout is fully responsive, adapting to the screen size of the device in use, so that the user experience is comparable on desktop computers, tablets, and mobile phones. This is important, as many participants access treatment materials via mobile devices. In one recent unpublished depression study, logins were nearly equally split between computers (2170 logins) and mobile phones (2035 logins).

### 5.2 The public homepage

**Fig. 2:**
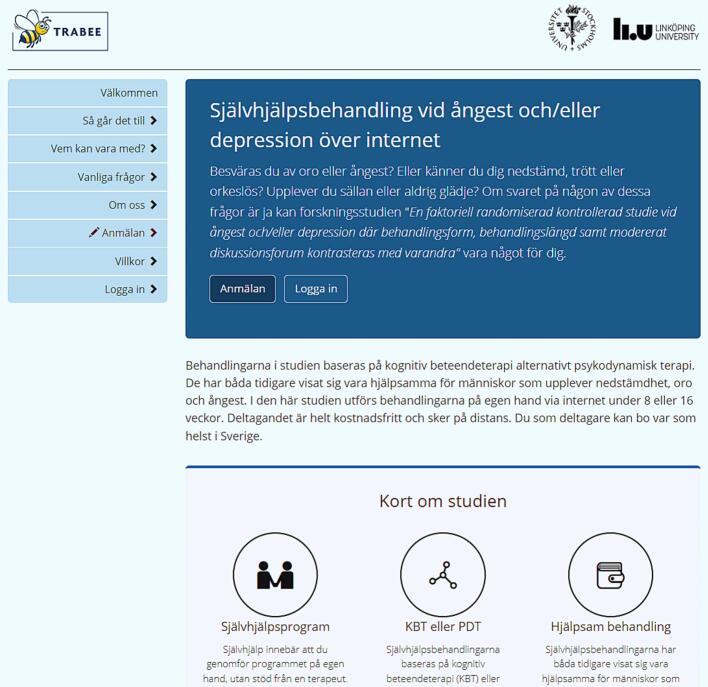
Screenshot of a study website: The TRABEE study, in Swedish, on depression and anxiety.

The public homepage is the first page visitors encounter (see Fig. 2 and more examples in Supplemental Fig. I, Supplemental Fig. II). It presents general information about the study: target group, treatment process, responsible researchers, and study staff. This page serves both as recruitment tool and as a way for help-seeking individuals to assess whether the study is relevant for them. The content can be edited directly by researchers.

### 5.3 The start page for logged-in users

**Fig. 3:**
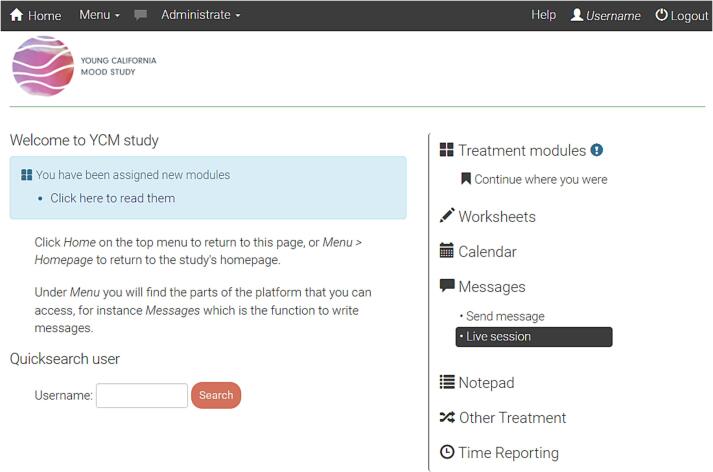
Screenshot showing the home screen (after logging in) for a participant on the normal-sized screen of a desktop computer or tablet.

Upon login, all users are presented with a start page for accessing available sections: treatment materials, message centre, worksheets, and other features (see Fig. 3 and one more example in Supplemental Fig. III). The same start page is shown to both participants and therapists, allowing therapists to see the participant perspective. Notifications about new events (new messages, newly available modules, pending questionnaires) are displayed in a notification centre at the top of the page. Therapists also see an administrative menu providing access to client data review, content administration, progress monitoring, and data export.

### 5.4 Treatment modules

**Fig. 4:**
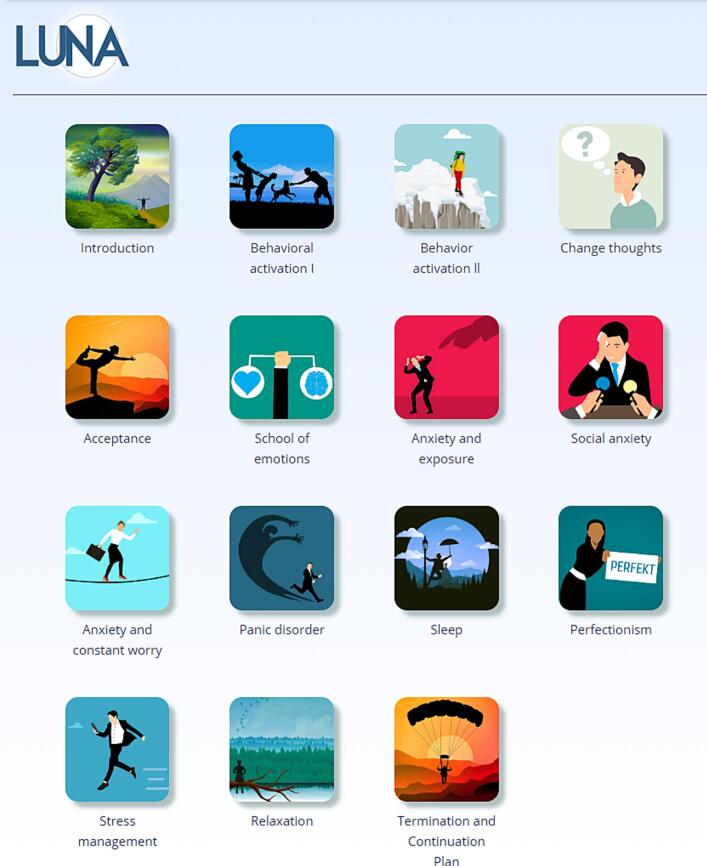
Screenshot showing the list of modules accessible to a user.

The primary section for participants contains the treatment materials, presented as formatted text, images, tables, embedded videos, audio files, linked PDF documents, and interactive worksheets (see Fig. 4 and more examples in Supplemental Fig. IV, Supplemental Fig. V). Materials are organised in a three-level hierarchy: branches, modules, and chapters.

A *branch* typically comprises all modules for an entire treatment programme (e.g., a CBT branch of eight modules). Because branches and modules can be assigned individually to participants, the platform supports tailored interventions, where treatment content is matched to each participant's symptom profile (Andersson, 2025). Each *module* consists of several *chapters* — pages of content presented sequentially. Module content is edited using a WYSIWYG editor.

Most interventions assign one module per week, but some provide two or more modules per week, including optional supplementary materials. Module assignment can be performed manually by therapists or automatically via predefined scheduling plans. In some interventions, participants choose their own modules from the available set using a drag-and-drop interface, based on brief descriptions of each module's theme. This approach to self-tailored interventions has shown comparable effectiveness to predetermined treatment sequences (Andersson et al., 2023).

### 5.5 Worksheets

**Fig. 5:**
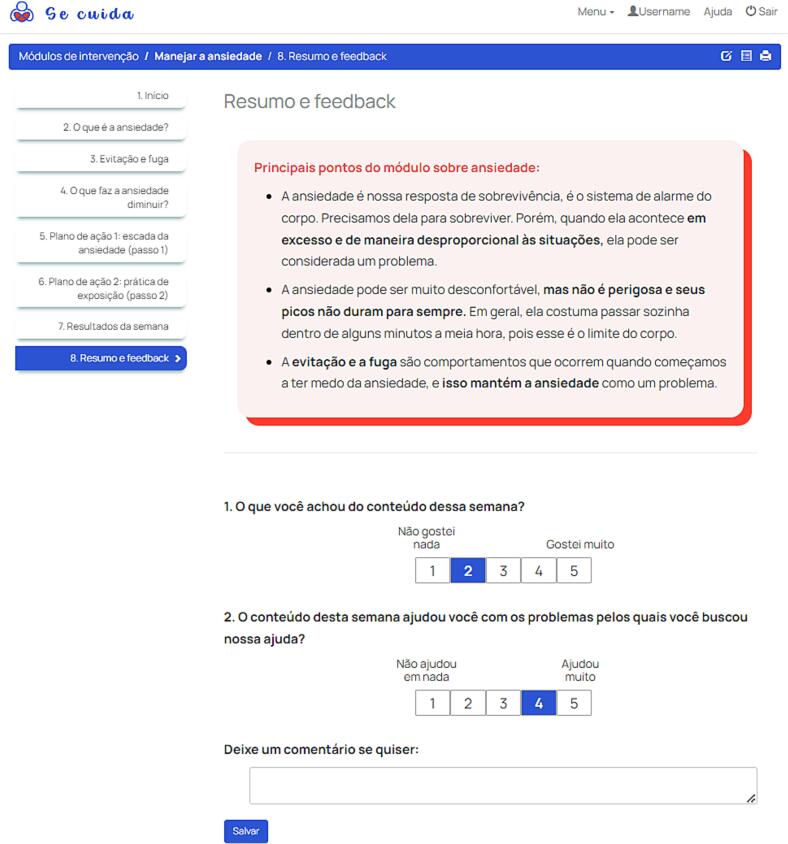
A worksheet page within a treatment module, from a study in Brazilian Portuguese about anxiety and depression.

An important component of many internet interventions are interactive exercises completed by participants during treatment. On Iterapi, these are implemented through worksheets, customisable interactive forms consisting of text boxes, checkboxes, buttons, sliders, and other input elements (see Fig. 5 and one more example in Supplemental Fig. VI). Worksheet data is saved and presented to therapists for review and feedback. Worksheets are highly customisable and can include straight forms, tables with variable numbers of rows, sliders, clickable images, multiple answer sets, references to previous answers, and conditional logic.

### 5.6 Messages

Communication between therapists and participants is supported through multiple channels: asynchronous messages, live text chat, audio/video chat, SMS, and, rarely, standard email.

Asynchronous conversations are the most common communication method. Messages are organised in threads based on topics (e.g., discussion of the current week's module). When a new message is posted, the recipient receives an email notification; however, message content is never included in notification emails, so all conversations remain accessible only within the secure platform environment. Additional features include file attachments, anonymous participant ratings of therapist response usefulness, and clinical supervisor ratings of therapist message characteristics.

Live text and audio/video chat is embedded entirely within the platform, without requiring external software. It is based on WebRTC technology, which provides end-to-end encryption for all communication sessions. Text chat conversations are saved and can be reviewed and exported for analysis. For high-security contexts, individual chat sessions can be deleted.

SMS is used for multiple purposes: sending one-time codes for multi-factor authentication, automatic questionnaire reminders, manual therapist-to-participant contact, and daily ecological momentary assessment (EMA) questions. The platform can send SMS messages to any country, supporting all character sets and emoticons.

### 5.7 Discussion forums

Discussion forums can be activated as a component of internet-based interventions, serving as a form of group interaction (Golkaramnay et al., 2007) or as an attention control condition. Access to forums is controlled through user groups, with therapists typically acting as moderators. Forums are organised in threads, with notifications sent when new posts appear. Forum access can be automated, for example with weekly access to new discussion threads.

### 5.8 Questionnaires and automatic assignments

Questionnaire administration is a central component of Iterapi. The system supports multiple question formats: free text, single and multiple choice, drop-down menus, matrix questions, date and time inputs, numerical inputs, ranking, computed equations, and conditional sub-questions. The layout is fully responsive, with larger formats such as matrices adapting to a linear format on smaller screens. Questionnaires can be designed from scratch via a user-friendly interface or copied (in whole or in part) from existing instruments.

Questionnaires can be assigned in several ways: manually by therapists, automatically during the registration process (as a screening component), or according to predefined schedules (e.g., weekly during treatment, post-treatment, and at follow-up intervals). Automatic reminders are sent via email or SMS if a participant has not responded within a specified number of days.

The platform automatically computes summary scores from individual responses, including total scores, sub-scale scores, and other calculated variables. These scores are displayed to therapists for rapid assessment and can be exported, along with all individual responses, to Excel files formatted for direct import into statistical software such as SPSS, R, or Jamovi.

### 5.9 Calendar and booking

A calendar component allows the creation of scheduled events involving one or more users, with automatic reminders sent via email, SMS, or screen notification. This is useful for planning chat sessions, interviews, and other scheduled interactions.

A complementary booking function allows participants to select an appointment from a pool of therapist-provided available times. This is used extensively, particularly during screening, where participants who meet inclusion criteria are immediately presented with available interview times. After booking, calendar events are automatically created for both therapist and participant, with reminders sent before the scheduled time (see Supplemental Fig. VII).

### 5.10 Ecological momentary assessment

Studies on the platform can incorporate ecological momentary assessment (EMA) through daily questions sent at predefined times (fixed, random, or both) via email, SMS, or screen notification (Shiffman et al., 2008). Responses are presented graphically to both participants and therapists and can be exported for analysis (see Supplemental Fig. VIII).

## 6 Administrative and therapist functions

### 6.1 User groups

Users are organised into groups (e.g., “Treatment group,” “Control group,” “Therapist X's clients”) that facilitate administration and data management. Group membership can be assigned manually or automatically, for example based on questionnaire scores meeting predefined cut-off values. Groups serve as a powerful filtering mechanism for data export, task assignment, and user management.

### 6.2 Performing operations with users

A range of operations can be performed with users: assigning roles and access rights, assigning questionnaires and worksheets, allocating therapists, placing users in groups, and randomising. These operations can be performed on individual users, user groups, or multiple selected users simultaneously. All operations can also be triggered automatically by the system based on predefined schedules or specific events (e.g., a participant completing a questionnaire, booking a meeting, or opening a treatment module).

### 6.3 Client journal

Each user has an associated log comprising automatically recorded actions (logins, module access, questionnaire completions) and manual entries written by therapists. The manual entries function as a client journal where therapists document interview notes, diagnostic assessments, and clinical observations, accessible to all therapists assigned to that participant.

### 6.4 Managing user data

The platform provides structured access to all data generated by a user. A “user hub” page centralises all user information in tabs, from which all operations related to that user can be performed: viewing data, assigning modules and questionnaires, writing messages, initiating chat, booking meetings, creating calendar events, and following the progress (see Supplemental Fig. IX). Access control ensures that therapists can only see data for their assigned participants, while administrators have broader access as defined by project management.

### 6.5 Exporting data

All user data stored on the platform can be exported to Excel files, with strict access controls mirroring the platform's visibility rules. Exported files are formatted with one row per user, facilitating import into statistical software. Additional computed fields can be included: calculated scores, word counts for messages and chats, login counts by device type, number of messages read, and number of modules opened.

## 7 Configuration and advanced features

### 7.1 Language versions and time zones

**Fig. 6:**
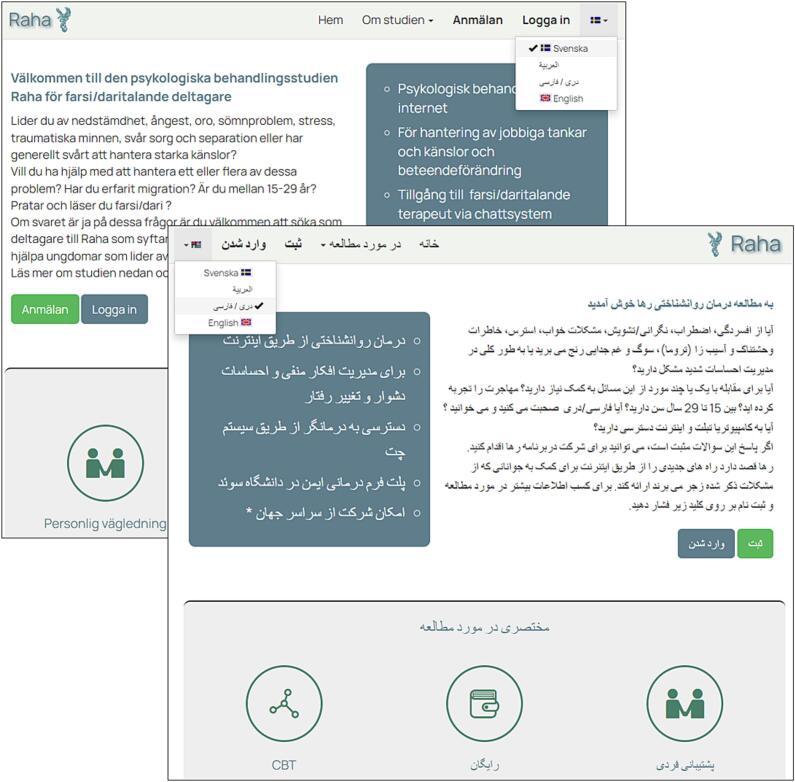
Two screenshots showing the easy change of the language version of a study homepage.

The entire platform interface (menus, instructions, and all automated communications) has been translated into more than 20 languages. For multi-language projects, users can switch languages on the fly or be locked to a chosen language upon registration. The platform automatically detects the visitor's browser language and displays the corresponding version. Therapists can review participant data in their own preferred language regardless of the participant's language setting. Full time zone support allows all dates and times to be displayed according to each user's local time zone (see Fig. 6).

### 7.2 Secure login and electronic identification

Iterapi provides multi-factor authentication (MFA) through SMS-based one-time codes as the second login step. SMS messages can be sent to any country, enabling MFA for all studies regardless of participant location.

In addition, the platform supports electronic identification through BankID, a widely used Swedish digital identification service utilised by 94% of smartphone users in Sweden in 2022. BankID authentication has been well received by participants in Swedish studies, providing a familiar and trusted login experience. The platform's architecture allows for integration of additional electronic identification services as they become available in different countries.

### 7.3 Automations

Automating the process flow that participants follow during a study has become increasingly important. Participants can be automatically included or excluded based on screening scores, with arbitrarily complex inclusion criteria evaluated immediately after screening. Inclusion can be followed by additional automatic operations: randomisation, therapist assignment, booking of screening interviews, assignment of treatment access, and scheduling of all assessment time points.

Automations can be triggered by various participant actions: registration, questionnaire completion, worksheet submission, booking of interview times, and module access. As noted earlier, at least two studies, including one with over 2400 participants, have run entirely automatically without scheduled therapist intervention.

### 7.4 Randomisation

The platform supports randomisation of participants to different intervention branches, with accompanying automatic actions based on group allocation: customised messages (e.g., treatment versus waitlist notifications), therapist assignment, module access, and questionnaire schedules. Randomisation can be performed at any point, whether after recruitment is complete or on a rolling basis, either manually or automatically following the screening process.

### 7.5 Anonymity and the dark web

The platform supports full participant anonymity through deployment on the Tor network. This functionality has been used in studies addressing sensitive topics where participants require assurance that their identity and location cannot be determined (see Section 3.6 for details).

### 7.6 Generic therapists

In most projects, participants are allocated to a named therapist. However, some study designs involve feedback from generic therapist accounts (e.g., “Your therapist”), behind which multiple therapists can operate interchangeably. This approach allows continuity for the participant while accommodating part-time schedules, holidays, and staff changes. Generic accounts function in parallel with personal accounts, so therapists can work with different participants in both personal and generic capacities.

### 7.7 Patient safety and monitoring critical client status

Patient safety in digital interventions has become a central concern, with recent evidence indicating that a substantial proportion of internet intervention studies do not systematically collect or report safety data. On Iterapi, safety monitoring is addressed through multiple mechanisms.

For interventions targeting populations with serious conditions (e.g., severe depression with suicidal ideation), automatic notifications are sent to therapists when critical situations are detected. These are triggered by specific response patterns, for example elevated scores on items assessing suicidal ideation or total scores exceeding clinical thresholds (e.g., scoring above 20 on the PHQ-9). Notifications can be delivered via email, SMS, and the platform's notification centre. The platform's automatic logging of participant activity (login frequency, module access, questionnaire completion) also enables therapists to identify patterns that may indicate deterioration or disengagement, which is particularly important for populations at elevated risk.

### 7.8 Alliance

One aspect of internet interventions is the *digital therapeutic alliance*, the extent to which platform design supports the development of a working relationship between participant and therapist (or between participant and the intervention itself). Evidence suggests that alliance ratings in digital contexts can approach those seen in face-to-face therapy, and that they are positively associated with treatment outcomes (Kaiser et al., 2021). As automated and virtual-therapist-driven interventions become more prevalent, measuring alliance toward non-human therapeutic agents has become a methodological priority, with dedicated instruments now emerging for this purpose (Miloff et al., 2020). Platform features that facilitate alliance, such as therapist messaging, personalised feedback, and timely communication, are increasingly recognised as active ingredients in their own right. Iterapi's support for multiple communication modalities (asynchronous messaging, live chat, video) positions it to support alliance-building across different clinical contexts.

### 7.9 Cultural adaption

Another aspect is the distinction between linguistic translation and true cultural adaptation. While Iterapi's support for more than 20 languages enables international deployment, effective cultural adaptation requires more than interface translation; it involves modifying treatment content, examples, and therapeutic approaches to align with local cultural norms, values, and healthcare contexts. Studies conducted on Iterapi in countries such as Lithuania, Romania, Japan, Brazil, and Turkey have involved varying degrees of cultural adaptation. Notably, a series of studies has specifically examined culturally adapted ICBT for immigrant populations within Sweden, including Arabic-speaking (Demetry et al., 2026) and Kurdish-speaking (Lindegaard et al., 2019) participants, demonstrating that the platform can serve as a vehicle for reaching underserved populations when treatment content is adapted beyond translation. Nonetheless, systematic frameworks for cultural adaptation in platform-based interventions remain an area of active development and also in light of AI and automatic translation tools.

## 8 Artificial intelligence integration

### 8.1 The evolving role of AI in internet interventions

AI, particularly large language models (LLMs), has opened new possibilities for internet-based psychological interventions (Carlbring et al., 2023). AI components have been proposed for multiple functions: generating therapeutic feedback, personalising treatment content, predicting treatment outcomes, detecting early warning signs, and providing scaled-up support in self-guided programmes. It can also be used for personalised outcome assessments (Garcia et al., 2025). The evidence base for AI-supported mental health interventions is growing, with recent meta-analyses suggesting that conversational AI and chatbot interventions can produce small to moderate effects on symptoms of depression and anxiety (Li et al., 2023), although concerns about safety, therapeutic alliance (Carlbring et al., 2023), and the handling of crisis situations remain (Torous et al., 2025).

A fundamental tension in this field is between scalability and transparency. Fully automated AI interventions can reach large populations at low marginal cost, but they often operate as “black boxes” where clinicians and researchers cannot inspect the specific responses generated for individual participants. For platforms serving a research function, where understanding the mechanisms of change is as important as achieving clinical outcomes, this opacity is problematic.

### 8.2 Conversation classification

The first AI-related feature implemented on Iterapi is a conversation classification system. Each message sent by a therapist is categorised into predefined categories: feedback on a specific worksheet, response to a health-related question, response to a technical question, and so forth. These classified conversations are then exported in a structured format that can be used to train AI models to generate feedback templates based on participant input. This classification system serves dual purposes: it provides structured data for AI training and, independently, enables research on therapist communication patterns and adherence to treatment protocols.

### 8.3 The compartmentalised architecture for AI integration

Rather than embedding AI directly into the treatment platform, which would require sending potentially sensitive participant data to external AI services, Iterapi has adopted what we term a *compartmentalised architecture*. This design separates AI processing from the treatment environment through distinct stages (see Table 2).

**Table 2:** Compartmentalised architecture in Iterapi.

1. Data export with de-identification	Participant data relevant for AI processing (e.g., questionnaire responses, treatment progress indicators) is exported from Iterapi in a de-identified format, with all personally identifiable information stripped.
2. External AI processing	The de-identified data is transferred to a separate, secure server environment where AI models process the data and generate outputs (e.g., personalised feedback, treatment recommendations, risk assessments).
3. Review and re-integration	The AI-generated outputs are reviewed by therapists using a predefined quality check, before being imported back into Iterapi and made available to participants. Hence, there is always a human clinician in the loop.

This architecture addresses several concerns. It maintains compliance with GDPR data minimisation principles by ensuring that AI processing operates only on the minimum necessary data. It preserves human oversight, as AI outputs pass through a review stage before reaching participants. It avoids vendor lock-in to specific AI providers, since the external processing component can utilise any AI service or locally hosted model. And it aligns with the transparency requirements anticipated under the EU AI Act for high-risk AI systems in healthcare.

### 8.4 Current implementation and ongoing research

The compartmentalised architecture is currently being applied in an ongoing study examining AI-enhanced feedback for social anxiety treatment, conducted as a collaboration between Stockholm University and Linköping University. In this study, participant progress data is exported from Iterapi to a separate national server that generates personalised feedback using LLM-based processing, which is then reviewed by therapists before being delivered to participants within the platform.

The platform's architecture could readily support direct AI integration through API keys, enabling real-time, automated feedback generation and personalised reminders without the export/import cycle. This approach is technically straightforward. However, we have deliberately chosen the compartmentalised approach for the following reasons: (a) it provides stronger data protection guarantees, as no raw participant data leaves the platform's secure environment in identifiable form; (b) it maintains a clear audit trail for AI-generated content; and (c) it ensures that the pace of AI integration is governed by evidence and clinical judgment rather than by technical capability alone.

### 8.5 AI components versus AI-driven therapy

Our approach reflects a broader position on AI's role in internet-based interventions. We distinguish between *AI components* (discrete functions where AI assists specific aspects of treatment under human supervision) and *AI-driven therapy*, where AI systems autonomously conduct therapeutic interactions. The former approach, which Iterapi pursues, allows for incremental integration of AI capabilities as evidence accumulates, while preserving the therapeutic framework and human oversight that guided ICBT is built upon. The latter approach, exemplified by standalone chatbot interventions, may offer advantages in scalability but raises concerns about therapeutic safety, the handling of clinical deterioration, and the difficulty of ensuring that AI responses are consistent with evidence-based treatment principles (Torous et al., 2025).

This distinction has practical consequences. The shutdown of Woebot, a pioneering AI therapy chatbot, in mid-2025, attributed in part to regulatory uncertainty around autonomous AI-driven mental health tools, illustrates the risks of moving ahead of both the evidence base and the regulatory framework. As AI capabilities continue to advance, the choice between component-based and autonomous AI integration will shape the next generation of internet interventions and the platforms on which they are delivered.

## 9 Future directions

### 9.1 Mobile applications

Iterapi is a fully responsive web-based platform, and all features function on mobile devices through web browsers. Notifications are delivered via email, SMS, or on-platform alerts. However, native mobile applications offer advantages in notification handling, offline access, and integration with device sensors. While we have explored mobile app development, the resource requirements for building and maintaining multi-platform, multi-language native applications are substantial, and the added clinical value relative to a well-designed responsive web platform remains uncertain. This question is under active discussion, and a progressive web app (PWA) approach, which combines web-based delivery with app-like notification capabilities, may offer a pragmatic middle ground.

### 9.2 Digital phenotyping and wearable integration

The proliferation of consumer wearable devices (smartwatches, fitness trackers) and smartphone sensors creates opportunities for passive data collection that could complement the self-report measures currently used on Iterapi. Digital phenotyping, the quantification of human behaviour through personal digital devices (Torous et al., 2016), could provide continuous, objective measures of sleep patterns, physical activity, social interaction, and physiological stress indicators. Integrating such data streams into the platform would enable richer characterisation of treatment response and could support just-in-time adaptive interventions. Technical and ethical challenges remain, particularly around data volume, participant burden, and informed consent for continuous monitoring.

### 9.3 Just-in-time adaptive interventions (JITAIs)

Just-in-time adaptive interventions deliver support precisely when it is most needed, based on real-time assessment of an individual's state and context (Nahum-Shani and Murphy, 2026). The combination of Iterapi's existing EMA infrastructure, potential wearable data integration, and AI-based processing could enable JITAI functionality, for example delivering a brief intervention module or coping prompt when sensor data or EMA responses indicate elevated distress. This represents an evolution from the current module-per-week delivery model and is an active area of development. However, as very brief interventions may be less effective than the standard length of ICBT programmes (Kaveladze et al., 2025) more research is needed.

### 9.4 Predictive analytics and machine learning

The platform's extensive database of treatment data, spanning thousands of participants across diverse conditions and countries, is a useful resource for developing predictive models. Machine learning approaches could be applied to predict treatment response, identify early indicators of dropout or deterioration, and optimise treatment sequencing. Previous work using Iterapi data has already explored data mining techniques for predicting tinnitus treatment outcomes (Rodrigo et al., 2021). Scaling these approaches while maintaining data protection could be a priority.

### 9.5 Gamification and engagement

Dropout and non-adherence remain common problems in internet-based interventions, with a recent meta-analysis reporting that approximately 25–30% of participants do not complete treatment (Karyotaki et al., 2015). Gamification elements (points, achievements, progress visualisation, and social comparison features) have been proposed as strategies to enhance engagement, though evidence for their effectiveness in clinical contexts is still emerging (Cheng and Ebrahimi, 2023). The platform's modular architecture allows for the integration of such elements within specific studies, enabling controlled evaluation of their impact on adherence and outcomes.

### 9.6 Regulatory developments

The regulatory environment for digital mental health interventions is changing rapidly. Germany's DiGA framework, which allows certified digital health applications to be prescribed and reimbursed through statutory health insurance, surpassed one million prescriptions by the end of 2024 and represents a model that other European countries may adopt. The EU Medical Devices Regulation (MDR; Regulation 2017/745), with its classification rules for medical device software, and the UK's NICE Evidence Standards Framework for digital health technologies further shape the requirements that platforms must meet. In December 2025, the European Commission proposed amendments to simplify MDR software classification, signalling that the regulatory framework continues to evolve. While Iterapi's primary function remains research, as research platforms and clinical tools converge, regulatory awareness must be part of platform development from the start.

## 10 Conclusions

The field of digital mental health platforms has expanded considerably since 2016, and the choice of platform for delivering internet-based interventions has become a methodological decision with implications for transparency, data governance, and the generalisability of research findings.

The Iterapi platform has evolved substantially since its original description in 2016. Over the past decade, it has supported more than 100 published research studies across at least 20 countries, contributing to the evidence base for internet-based interventions targeting more than 35 clinical conditions, from common problems such as depression and anxiety to less-studied areas including climate change distress, loneliness, and problem gambling. The platform has also expanded beyond its CBT origins to support psychodynamic therapy, acceptance and commitment therapy, mindfulness-based interventions, and virtual reality studies (Andersson, 2025).

The developments described in this update reflect the field's broader evolution. Security and regulatory compliance have moved from background concerns to central design requirements, with GDPR compliance and emerging AI regulations shaping platform architecture. The proliferation of competing platforms, commercial, academic, and open-source, has made the choice of treatment platform a methodological decision worthy of explicit justification in research protocols. The rapid advancement of AI capabilities has required careful consideration of how to integrate these technologies while preserving transparency, privacy, and human oversight.

Our approach to AI integration, from conversation classification through compartmentalised cloud processing to the prospect of locally hosted language models, illustrates a broader principle: that technical capability should be governed by clinical evidence and ethical consideration rather than the reverse. The platform *can* integrate AI directly; we have chosen not to, until the evidence supports doing so safely and effectively. This cautious approach, combined with transparency and researcher control, positions Iterapi to adapt as the field evolves.

Developing and maintaining a platform for internet interventions is a continuous process. Close communication between developers and researchers, supported by university infrastructure, ensures that the platform can respond to new clinical needs, technological opportunities, and regulatory requirements. The platform's multilingual support and international collaborations provide a basis for the large-scale, cross-cultural implementation research that the field now needs.

## CRediT authorship contribution statement

**George Vlaescu:** Software, Conceptualization, Writing – original draft. **Per Carlbring:** Writing – review & editing. **Gerhard Andersson:** Conceptualization, Supervision, Writing – review & editing.

## Declaration of Generative AI and AI-assisted technologies in the writing process

Generative AI tools were used during the preparation of this manuscript for proof reading. The authors reviewed and edited all AI-generated content and take full responsibility for the content of the publication.

## Funding

The Iterapi platform has been developed and maintained as research infrastructure at 10.13039/501100003945Linköping University, with institutional support from the university and project-specific funding from research grants awarded to the authors. Some collaborating research groups at other universities have made small contributions toward the IT system developer's time allocated to their specific projects, but no commercial entity has funded the platform, and no licensing fees are charged to academic users.

## Declaration of competing interest

The authors declare that they have no known competing financial interests or personal relationships that could have appeared to influence the work reported in this paper.

## Figures and Tables

**Supplemental Fig. I:**
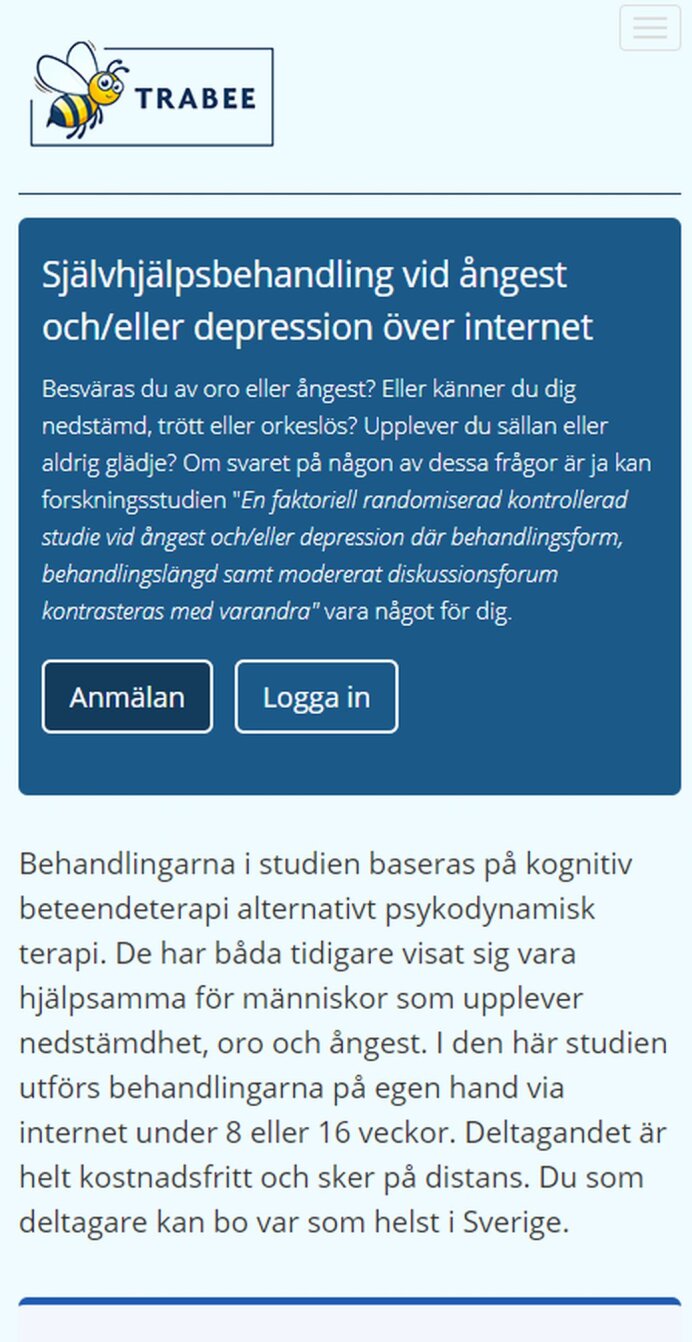
Screenshot of a study homepage on a mobile phone, demonstrating responsive layout.

**Supplemental Fig. II:**
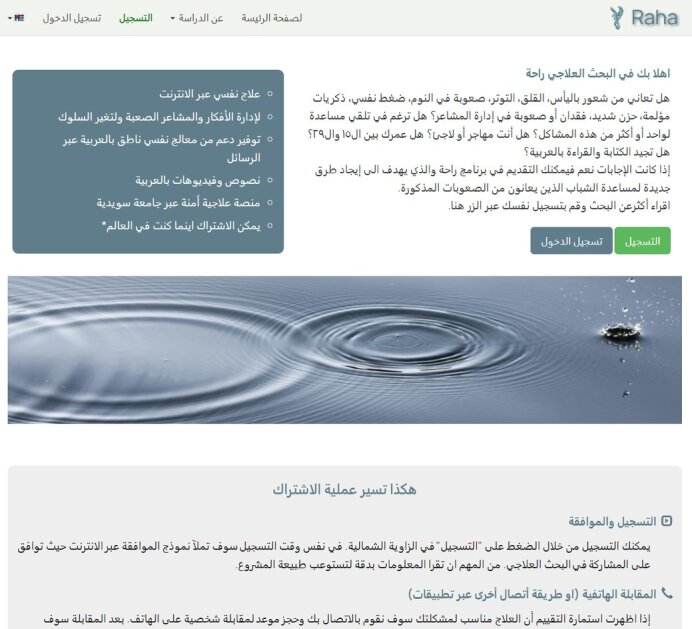
Screenshot of a study homepage in Arabic, showing a right-to-left layout.

**Supplemental Fig. III:**
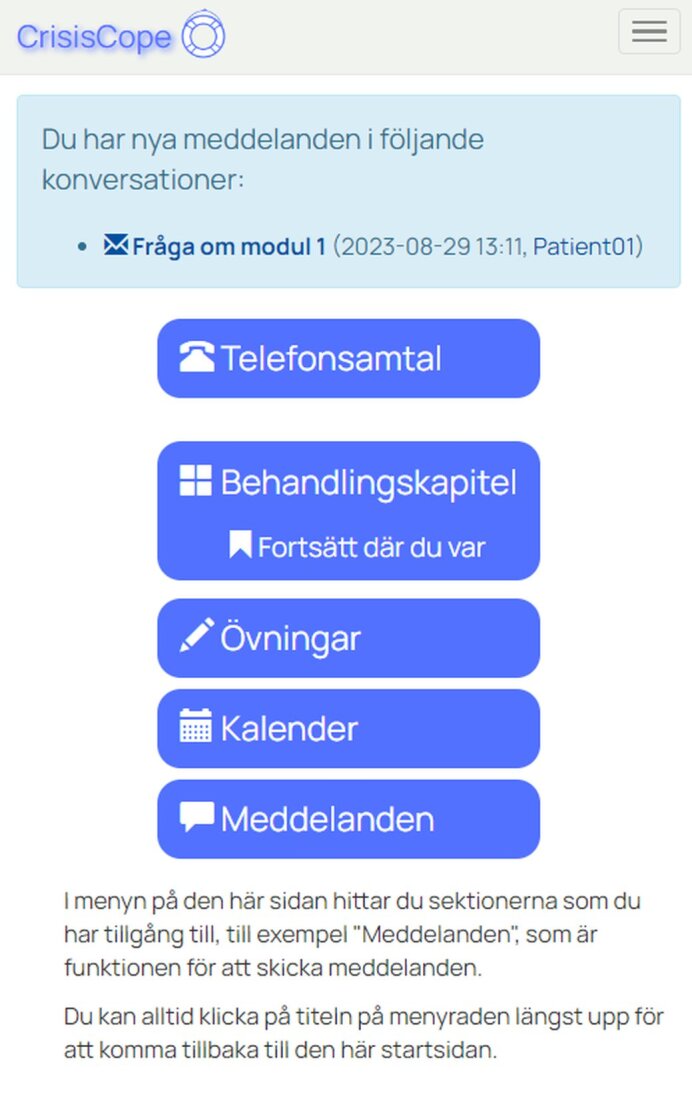
Mobile view of the participant start page after logging in, showing a notification message.

**Supplemental Fig. IV:**
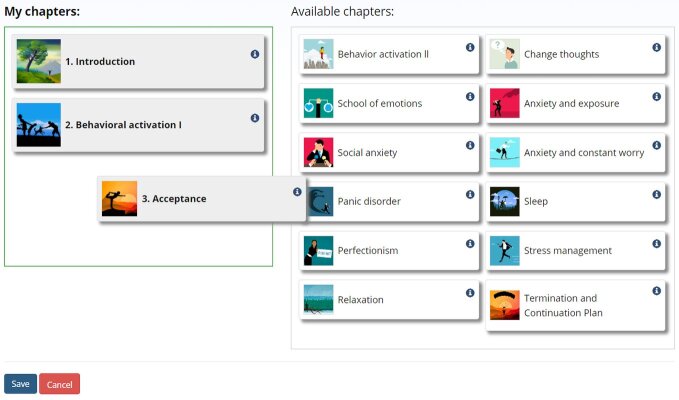
One of the various ways of choosing interventions modules for a participant, via dragging-and-dropping from a list.

**Supplemental Fig. V:**
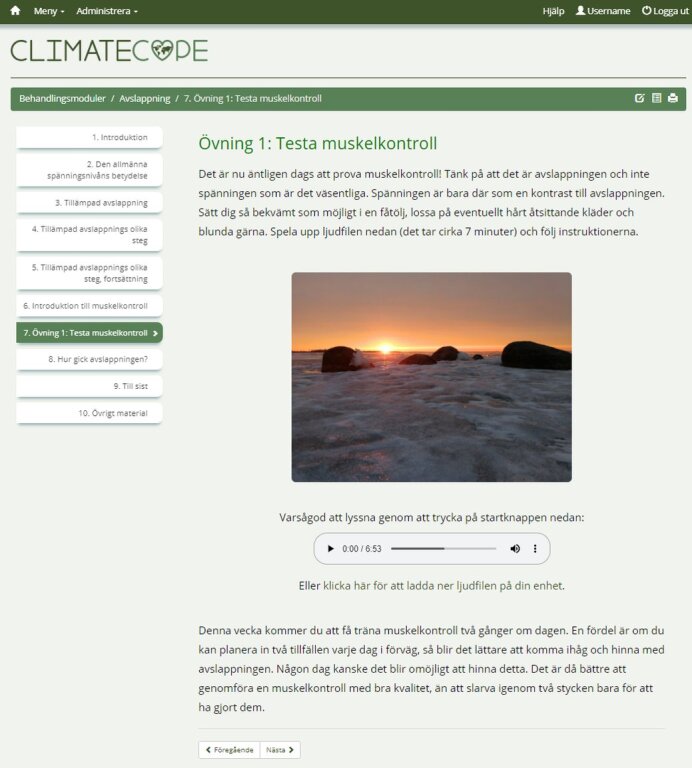
A page inside an intervention module, which may include images, embedded audio and video files and more.

**Supplemental Fig. VI:**
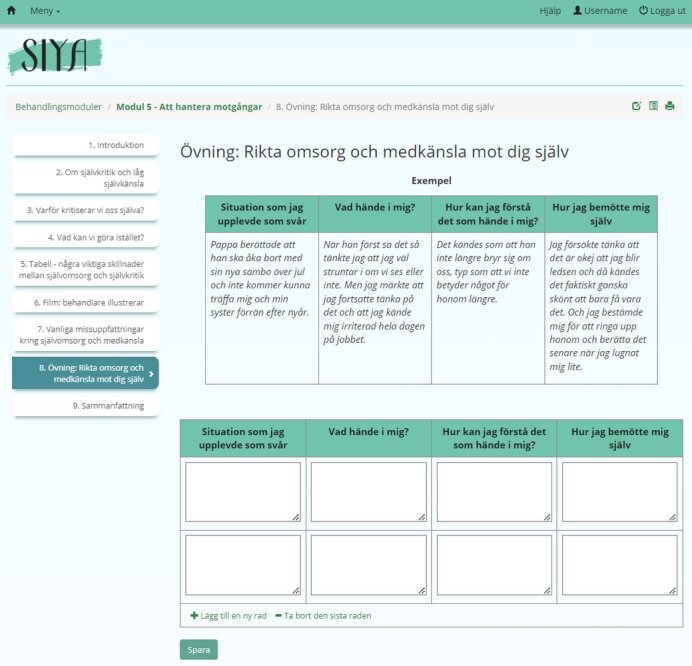
A module page containing an interactive worksheet where participants may fill in a table and add more rows.

**Supplemental Fig. VII:**
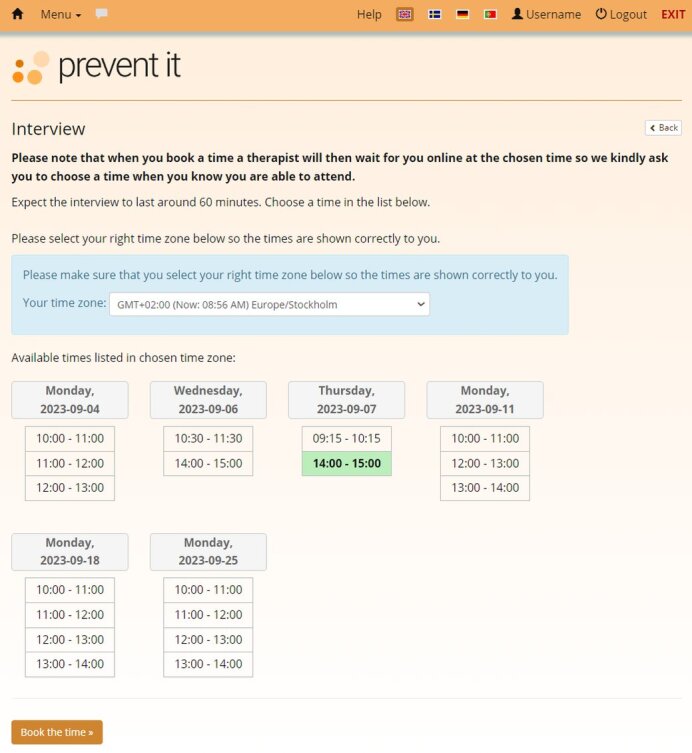
Screenshot when a participant books a time for an interview, in an international study where they may also choose their own time zone.

**Supplemental Fig. VIII:**
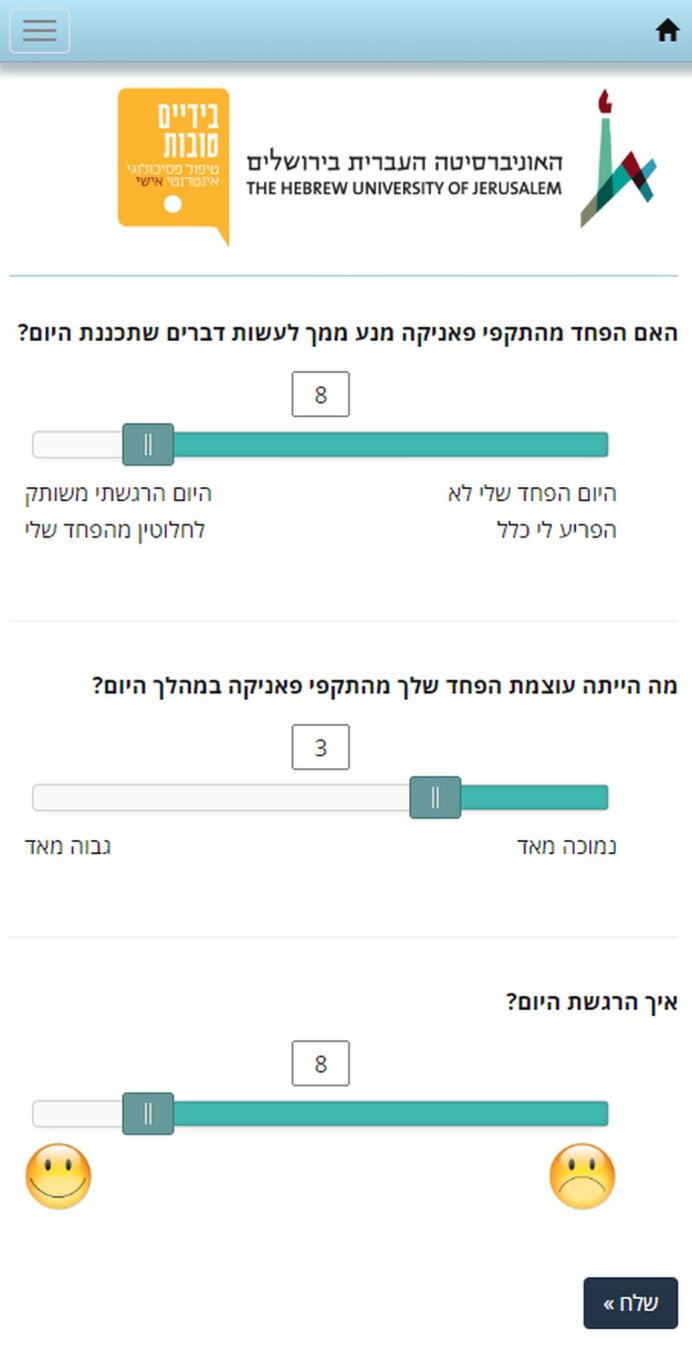
Screenshot of the daily questions (EMA) as shown to the user on a mobile phone, in Hebrew.

**Supplemental Fig. IX:**
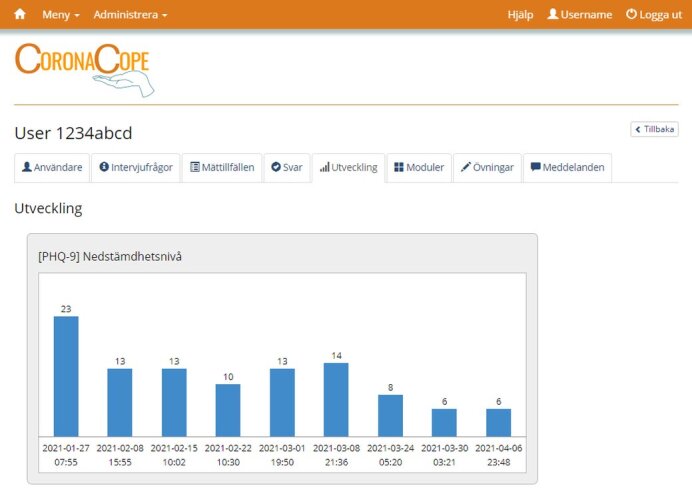
Screenshot of the therapist administrative view with the progress monitoring section.

## Data Availability

No data were used for the research described in this article.
